# Annotations

**Published:** 1907-07-06

**Authors:** 


### Annotations.

### Sir William Collins, M.D., M.P.

There never was a time in the history of medicine when the profession stood in greater need of an educated, resolute, strong mind to voice its affairs and represent it worthily in Parliament. It has been well said that, if a man possess character, education, and fearlessness in the maintenance of his opinions and the enforcement of the principles in which he believes, there is no position in the country which may not be open to him. One of the most honourable offices in connection with British universities is that of Vice-Chancellor of the London University. The constitution of the London University, the esteem in which its degrees are held as tests of knowledge and ability, and the independence and thoroughness of its senate, all combine to make the vice-chancellorship a high appointment of great authority. We heartily congratulate Sir William Collins, M.P., upon his acceptance of this office. He took his degrees with honours, and won the gold medal in public health in 1887. Sir William has done excellent work as a Fellow and Senator, and his public services as chairman of the London Education Committee were exceptionally great. As chairman of the London County Council he won golden opinions, and his election to the House of Commons conferred honour on the constituency which he represents. We have had considerable personal experience of Sir William Collins' marked ability, judgment and character in the discharge of public business, and we look forward to the time when he may render yet more important service not only to the profession, but to the country, by being selected as a member of a Liberal Cabinet. Sir William's record proves him to be a man who would bring strength to any Ministry he may join, not only from his personal qualities, but because of the robustness of his character, and his power to present his subject with a terse directness sure to command attention. The Senate of London University is to be congratulated upon its choice of a vice-chancellor, whilst Sir William Collins' election is a just tribute to his ability, and to his university and public record.

### Special Hospitals and their Visiting Staffs.

In our issue of the 15th ultimo, p. 274, we laid stress on the fact that special hospitals have greatly improved of late years, and that many members of their visiting staffs in London are amongst the best qualified and most honourable men in the profession. We are glad to emphasise this opinion by recording the results which have followed Mr. Francis Roe's letter, calling in question the conduct of a member of the visiting staff of a special hospital, the details of which we have already published. Mr. Francis Roe, it was fair to presume, had satisfied himself as to the accuracy of the statements made over his signature in the letter in question. It would appear, however, as if Mr. Roe had first made the charges and then investigated them, for he now writes: "I find that the facts were not as alleged, and therefore that my inferences were not correct. I have since seen the member of the staff of the hospital referred to, and, after hearing his clear statement as to what really occurred, I have no hesitation in publicly withdrawing the letter which I wrote, and asking him to accept my *amende honorable* for having written it." This letter is dated 26th ultimo, and we could have wished that Mr. Roe had more closely tested his facts before writing at all on a matter of profound importance not only to special hospitals and their visiting staffs but to the public at large. We are glad to know that the charges made, after full investigation, have been withdrawn, a fact from which it may fairly be held that the present administration of special hospitals in London is infinitely better than it was. Special hospitals have had a material influence for good upon the treatment of disease, and the relief of suffering humanity. The best of them fulfil a very useful purpose, and we congratulate all concerned that prompt action has secured a full refutation of the charges published by Mr. Francis Roe.

### The late Sir William Tennant Gairdner, K.C.B.

It is with sincere and profound regret that we record the death of Sir Wm. Tennant Gairdner. In him there passes away one of the great figures of the medical profession. A great physician and a good man is no more, and the world is indeed the poorer for the loss. For more than fifty years he was continuously engaged in the teaching and training of medical students, and it is in the lives and work of these men that his moral force and influence are most fully realised. Meet them where you will, their talk sooner or later comes round to the one topic, and is full of an affectionate reverence and tenderness for their old teacher. The measure of his professional achievements is much—it is to be found in his writings and in the sanitary history of the city where he lived and worked for well-nigh forty years. Much also might be said of the range of his knowledge, of his wide sympathies, and commanding ability. But it was something over and above these which placed him in the unique position he long occupied in the regard and judgment, not only of his juniors, but also of his compeers in the profession. The secret may perhaps be found in his character and personality. It was impossible to know Gairdner without becoming aware that he carried into everything with which he was concerned a sincerity and singleness of aim entirely beyond question, and that never was he moved by other than large and worthy motives—and this consistently and invariably, because, as it were, naturally and without effort. Add to this elevation of character, a certain simplicity of outlook and gentleness of spirit, and it is possible to get some measure of insight into the reverence and affection which Gairdner inspired. When, further, it is remembered that these mental and moral qualities were constantly exhibited in the class-room and the hospital ward as the natural attitude of the man towards his work and its various claims, it may be understood that to many Sir Wm. Gairdner was a source of inspiration not less than the object of a deep personal affection.

